# Relationship among service accessibility, social norms, herpes zoster vaccination intention of middle-aged and older adults in Chongqing: mediating role of perceived usefulness

**DOI:** 10.3389/fpubh.2025.1649455

**Published:** 2025-09-26

**Authors:** Yanxi Deng, Tianran Wang, Li Yu, Xin Fu, Yaman Luo, Fei Chen

**Affiliations:** ^1^Center for Medical and Social Development Research, School of Public Health, Chongqing Medical University, Chongqing, China; ^2^School of Public Health and Management, Guangzhou University of Chinese Medicine, Guangzhou, China

**Keywords:** aging, herpes zoster vaccination intentions, stimulus-organism-response theory, perceived usefulness, access, structural equation model

## Abstract

**Objective:**

The study aims to explore the relationship of vaccination service accessibility, social norms, and perceived usefulness on the intention to get the herpes zoster vaccine (HZV) among middle-aged and older adults, and to provide suggestions for promoting the health and disease prevention of this population.

**Methods:**

A questionnaire was developed based on the Stimulus-Organism-Response (SOR) theory. Utilizing stratified random and convenience sampling, the study enrolled participants aged 45 years and above. A structural equation modeling approach was employed to explore the relationships among external factors, perceived usefulness and vaccination intention among the surveyed population.

**Results:**

Ultimately, a datasets consisting of 481 valid samples was obtained. Social norms and perceived usefulness were positively associated with the intentions of middle-aged and older adults to receive HZV. Accessibility of vaccination services and social norms were also positively related to perceived usefulness, while accessibility showed no direct correlation with vaccination intention. Perceived usefulness was found to mediate the relationships between both accessibility and social norms with vaccination intention.

**Conclusion:**

Social norms and perceived usefulness are significant associated with vaccination intentions. Both vaccination services and social norms were related to vaccination intention through the mediation of perceived usefulness. This study emphasizes the role of perception in external information and willingness to vaccinate. Vaccination management should be strengthened to enhance service accessibility and reduce economic burdens. Promote a social consensus on vaccination to prevent disease. Attention to social norm contents and official channels, coupled with increased perceived usefulness, can further bolster vaccination intentions among adults aged 45 and above.

## 1 Introduction

The global incidence of herpes zoster (HZ) ranges from 3 to 5 cases per 1,000 person-years, with studies indicating a sharp age-dependent increase in incidence among adults aged ≥50 years (1). Some patients with HZ may develop postherpetic neuralgia (PHN), which seriously affects the patient’s quality of life and causes great stress and burden on the body and mind (2). With global population aging accelerating, middle-aged and older people’s immunity is gradually declining. This elevates their susceptibility to HZ—a condition associated with substantial disease burden and profound quality-of-life impairment (3). Promoting health in this population is critical to achieving. China’s “healthy aging” goals, particularly through evidence-based preventive measures such as vaccination. The World Health Organization (WHO) recommends expanding both the coverage and range of herpes zoster vaccine (HZV) included in immunization programs (4). Despite existing clinical guidelines recommending herpes zoster vaccination in several countries, vaccination coverage and acceptance remain suboptimal in key regions, including the United States (5), the United Kingdom (6), and multiple WHO member states (7). Consequently, public health initiatives to expand vaccination programs and improve immunization rates are critically needed, particularly targeting populations with persistently low vaccine uptake (8). The HZV approved for marketing in China has had major revisions due to advancements in clinical research and vaccine development. One such update is extending of the suggested age indication to adults aged ≥40 years (9). This study’s emphasis on adults over 45 is especially justified and relevant.

Previous research on willingness to receive HZV has primarily focused on individual-level factors, emphasizing intrinsic factors such as cognitive status (10, 11) and demographic characteristics (12, 13). However, vaccine intention is influenced by external social and environmental factors, including the availability of vaccination services (14) and social norm-driven recommendations (15). There is a significant positive correlation between perceived usefulness and the willingness to get influenza vaccines (16). This studies have expanded the conceptual understanding of vaccination behavior by identifying key determinants, collectively and profoundly highlighting the importance of exploring potential interactions among these factors. Specifically, individuals’ perceptions related to vaccines closely connected to external environmental influences. This raises critical questions about whether environmental factors are associated with intrinsic perceptions, and whether perception may mediate the relationship between external environments and vaccination behaviors. To comprehensively investigate how two key environmental stimuli—availability of vaccination services and social norms—relate to individual perceptions and eventually correlate with HZV intention, the study employs the Stimulus-Organism-Response (SOR) framework.

The SOR framework serves as a theoretical framework for analyzing the relationship between environmental stimuli, individuals’ cognitive-emotional processes, and subsequent behavioral responses. It suggested that the SOR model can better explain the dynamic mechanism of how external information is processed and how behavior are formed when analyzing willingness (17). Unlike the traditional stimulus–response (S-R) model, the SOR model introduces the crucial mediating role of the “organism,” emphasizing that external stimuli impact behavior by influencing an individual’s cognitive and affective states (18). The “organism” focuses on perception processes. According to the predictive processing framework, perception aims to represent the environment in terms of individuals’ action opportunities. Through perceptual inference, sensory outcomes are more readily translated into actions beneficial to the individual. In essence, perception guides people toward behaviors that maximize personal usefulness and value (19). So perceived usefulness is a critical dimension and an important observation of overall perceptions that directly informs health-related behaviors. Currently, the SOR model has been widely utilized in consumer behavior (20) and information science studies (21). Within the health domain, recent investigations have adopted this framework to explore behavioral intentions. For instance, researches have employed the SOR model to analyze how the quality and credibility of fitness-related social media content impact exercise intentions via perceived expertise and usefulness (22). Another study examined the model to explore vaccination decision, demonstrating how information sources (stimulus) affect parents’ intention to vaccinate their children against COVID-19 (response) through the mediating role of vaccine confidence (organism) (23). These studies demonstrate the model’s applicability in explaining vaccination-related behaviors within environments. We identify accessibility of vaccination services and social norms as the stimuli, perceived usefulness as the organic factor, and vaccination intention as the behavioral responses among middle-aged and older adults.

Accessibility refers to the ease with which individuals can meet their healthcare needs, primarily considering the convenience of reaching healthcare facilities and obtaining services (24). The four attributes of availability, accessibility, affordability, and acceptability (4A) are commonly employed to gage access (25). Vaccination services, as a fundamental component of healthcare, are subject to the same evaluative criteria regarding service accessibility. Based on the aforementioned analytical framework, the connotation of this study pertains to the degree of appropriateness and accessibility between the supply and demand of preventive vaccination services. The accessibility of preventive vaccination services serve as an external stimulus, measured through the subjective perception of the middle-aged and older adults. Population aging has increased the demand for disease prevention among middle-aged and older adults. Compared to child vaccination services, there are fewer vaccination institutions and vaccine types available for them, which has significantly impacted their vaccination uptake rates (26). Limited convenience in accessing vaccination facilities may result in low acceptance of these services, potentially generating barriers and negative perceptions toward vaccination (27), thereby diminishing vaccination willingness. Accordingly, the following hypothesis is proposed:

*H1*: Accessibility of vaccination services is positively related to HZV intentions among middle-aged and older adults.

Social norms are the widely accepted standards of behavior, values, and expectations within a society or group. Through acquisition, transmission, and transformation, these social norms become internalized in individual consciousness (28). We takes social norms as external stimulus variables. Prior research has demonstrated that outbreaks of herpes zoster enhance individuals’ willingness to receive the HZV (29), suggesting that behavior is influenced by herd mentality within particular social contexts. People internalize certain social norms as personal standards, improving their behavior either by adopting the practices of their social network majority or by avoiding perceived risks (30). Accordingly, the following hypothesis is proposed:

*H2*: Social norms are positively associated with HZV intentions in middle-aged and older adults.

Within the SOR framework, perceived usefulness functions as a critical “organism” variable, embodying both cognitive and emotional evaluative dimensions (31). Middle-aged and older adults engage in a rational cognitive evaluation of vaccination benefits by considering external factors such as vaccination service conditions and implicit social norms. Subsequently, the rational cognitive evaluation fosters emotional trust in the safety and effectiveness of vaccine, which further generates positive response—perceived usefulness. Trust encompasses confidence in both effectiveness and safety of vaccines (32). From the perspective of trust transfer theory, individuals with sufficient trust will perceive the behavior of vaccination as highly useful (33). Thus, perceived usefulness integrates rational assessments of benefits with emotional trust. In this study, perceived usefulness is defined as the degree to which middle-aged and older population regard vaccination as advantageous based on the rational judgment and emotional identification concerning the vaccine and its policy. The study found that the public’s perceived usefulness of influenza vaccines significantly affects their intention to get vaccinated. The priority for vaccination, awareness of vaccine function, and effectiveness of the vaccine are crucial items of perceived usefulness (16). Middle-aged and older adults who perceive vaccine as useful (safe and effective) are more likely to get HZV to mitigate the adverse consequences of the disease (34).

Through the vaccination services they receive, middle-aged and older adults cultivate a sense of belief and positive attitudes. Conversely, limited accessibility to vaccination services may pose challenges for middle-aged and older adults, potentially undermining positive beliefs and leading to vaccine refusal (35). When evaluating the perceived usefulness of health-related actions, individuals often seek social validation from their peers before adopting such behaviors. In terms of alcohol protection, existing studies have shown that perceived usefulness mediates the relationship between social norms and individuals’ willingness (36). Vaccination, as a type of health behavior, may exhibit the same association of perceived usefulness between social norms and willingness. Social norms can help middle-aged and older adults generate and intensify perceptions about HZV and themselves, and help them make more reasonable and feasible decisions under limited conditions. The main access to information related to HZV for the people is medical personnel, family and friends, and the media (37). The vaccination status of close contacts and the perspectives of family, friends, and medical personnel (38) constitute social norms that impact individual value perceptions and, consequently, the willingness to receive HZV. Based on these observations, the following hypotheses are proposed:

*H3*: Perceived usefulness is positively related to HZV intentions in middle-aged and older adults.

*H4*: Accessibility of vaccination services is positively related to the perceived usefulness of HZV in middle-aged and older adults.

*H5*: Social norms are positively related to perceived usefulness of HZV in middle-aged and older adults.

*H6*: Perceived usefulness of HZV plays a mediating role in the accessibility of vaccination services and vaccination intention.

*H7*: Perceived usefulness of the HZV plays a mediating role in social norms and vaccination intention.

## 2 Materials and methods

### 2.1 Study design

Taking Chongqing as an example, we implemented a mixed sampling approach to balance methodological rigor with practical feasibility. At the regional level, we employed stratified sampling based on the per capita GDP levels reported by the local statistical bureau in 2022 (39) and the World Bank’s per capita GDP data for 2023 (40). The study first stratified districts into two groups of high-income and upper-middle-income. Within each selected group, two districts/counties were randomly selected. Subsequently, one to two streets/townships were randomly selected from each chosen district/county, and one to three communities/villages were randomly selected from each street/township. In total, the survey was conducted across seven streets/townships. At the individual level, participants aged 45 and above were recruited through convenience sampling within these selected communities/villages. Ensuring the suggestion that the ratio of the number of sample sizes to the number of latent variable measures is greater than 10:1 (41). All participants were at least 45 years old, lived in Chongqing for more than half a year, and were voluntary and conscious to participate in the survey. A final sample size of 481 was obtained, with an effective rate of 93.94%. The basic demographic characteristics of the 481 participants are shown in Table 1.

**Table 1:** Demographic characteristics of the participants (*N* = 481).

Characteristic		Number (%)	Characteristic		Number (%)
Sex	Male	224 (46.6)	Monthly disposable income (CNY)	≤2,000	156 (32.4)
	Female	257 (53.4)		2,001–4,000	130 (27.0)
Age (years)	45–59	328 (68.2)		4,001–6,000	90 (18.7)
	60–74	114 (23.7)		6,001–8,000	54 (11.2)
	≥75	39 (8.1)		>8,001	51 (10.7)
Educational level	Primary	115 (23.9)	Self-assessed health status	Very good	101 (21.0)
	Junior high schools	112 (23.28)		Good	212 (44.1)
	Senior high schools	103 (21.4)		Fair	144 (29.9)
	Junior colleges and above	151 (31.4)		Poor	22 (4.6)
				Very poor	2 (0.4)
Chronic disease (types)	0	349 (72.6)			
	1	95 (19.8)			
	≥2	37 (7.7)			

### 2.2 Questionnaire design

The content of the questionnaire included demographics, accessibility of vaccination services, social norms and vaccination intention. The vaccination intention and other variables were measured with a 13-item scale. The scale was measured using a 5-point Likert scale, with scores ranging from 1 to 5 in order from strongly disagree to strongly agree.

#### 2.2.1 Demographic characteristics

The first part of the questionnaire was demographic information such as sex (male, female), age (45–59 years, 60–74 years, ≥75 years), educational level (primary and junior high schools, senior high schools, junior colleges and above), chronic disease (0, 1, ≥2), monthly disposable income (≤2,000, 2,001–4,000, 4,001–6,000, 6,001–8,000, >8,000), self-assessed health status (very good, good, fair, poor or very poor).

#### 2.2.2 External stimulus of vaccination intention

The key variables of stimulus including accessibility of vaccination services and social norms. Accessibility of vaccination services consisted of four items, social norms consisted of three items. The availability of vaccines, the convenience of approaching a vaccination institution, the affordability of the cost of vaccination, and the acceptance of vaccination services were all given average scores based on the previous definition of accessibility of vaccination services (14, 25). Furthermore, inadequate access to vaccine-related information emerged as a significant barrier to HZV uptake (42). Our adapted framework incorporates information availability as a predictive item of availability. We define social norms as the respondent’s approval regarding the opinions of the surrounding population. This average score obtained from three dimensions: the consideration of opinions from family members, relatives and friends; the consideration of opinions from medical professionals; and the consideration of the behavior recognized by the majority in society.

#### 2.2.3 Perceived usefulness

As previously illustrated, safety and usefulness are key elements of perceived usefulness (34). Given that perceived usefulness in this study reflects an affective evaluation, the overall judgment of vaccination benefits to the individual was also incorporated. To enhance scientific rigor and practical applicability of the measurement items, we adapted the confidence dimension items from the 5C scale (43) and its Chinese version (44), modifying and adding them to align with the context of HZV. The final perceived usefulness score was computed as the average of safety, effectiveness, confidence, and benefit ratings.

#### 2.2.4 Vaccination intention

We reviewed previous studies related to vaccination intentions. A comprehensive review of the extant literature reveals that the majority of studies have employed the term “intention to vaccinate” as a metric for measuring vaccination intention. In contrast, a smaller number of studies have incorporated the concept of “willingness to vaccinate partners” as an additional dimension in the analysis of vaccination intention (27). Recommended HZV vaccination intentions among middle-aged and older adults were also included in the analysis of intentions from a peer support perspective (45). Consequently, we expanded the intention dimension to encompass the variable of “willingness to recommend HZV to others” to assess the comprehensive evaluation of HZV vaccination intention among middle-aged and older adults.

### 2.3 Questionnaire validation

Following the questionnaire’s initial creation, the group engaged in multiple discussion and revision on it within 5 weeks. Additionally, we gathered pertinent feedback from two medical statistics specialists, four health management specialists, and three public health specialists. Incorporating their recommendations, the economic affordability component within the accessibility dimension was explicitly refined to encompass costs such as transportation fees and service charges, while also integrating information related to the availability of vaccination services. After that, 32 middle-aged and older adults over 45 participated in a pre-survey. The questionnaire’s wording and structure were once more adjusted according to the survey’s actual circumstances. The results indicated that the reliability and validity of the questionnaire met the required standards, and the division of dimensions was reasonable. Further refinements were made based on pretest findings, including repositioning items of intention to the final section of the questionnaire, adding explanatory notes on the colloquial term for herpes zoster, and removing redundant phrasing enhance clarity and conciseness. The question items of each dimension of the scale are shown in Table 2.

**Table 2:** Content of questionnaire variables.

Variables	Items	Question	Source
Accessibility of vaccination services (VA)	VA1	I can get services such as herpes zoster vaccine and related information	Lu et al. (14), Zeng and Ling (25)
	VA2	It’s convenient for me to go to the Vaccination institutions	
	VA3	The cost of herpes zoster vaccine is affordable for me (including the cost of vaccination, services, transportation, etc.)	
	VA4	I can accept the herpes zoster vaccines offered	
Social norms (SO)	SO1	I will consider the advice of my family, relatives and friends on whether or not to get vaccinated	Betsch et al. (43), Zhang et al. (53)
	SO2	I will consider the advice of medical professionals on whether or not to get vaccinated	
	SO3	I’ll consider getting the herpes zoster vaccines when most of the people around me are vaccinated against them	
Perceived usefulness (PU)	PU1	I am completely confident that herpes zoster vaccines are safe	Zhang et al. (44), Gao et al. (45)
	PU2	Herpes zoster vaccines are effective	
	PU3	Regarding vaccines, I am confident that public authorities decide in the best interest of the community	
	PU4	Overall, I think the herpes zoster vaccines are beneficial to me	
Vaccination intention (VI)	VI1	I’m willing to get herpes zoster vaccines	Lu et al. (27), Gong et al. (42)
	VI2	I would recommend the herpes zoster vaccine to others	

*VA, accessibility of vaccination services; SO, social norms; PU, perceived usefulness; VI, vaccination intention.

The internal consistency of each dimension was verified by the Cronbach’s α coefficient reliability test, and the total Cronbach’s α coefficient of the scale was 0.925. The Cronbach’s α coefficients of each dimension were above 0.8. The KMO value of the questionnaire was 0.900, and Bartlett’s test of sphericity (*χ*^2^ = 4452.176, *p <* 0.001). The total variance explained by the rotation of the four factors was 79.055%. Factor loadings, Composite Reliability (CR) values, and Average Variance Extracted (AVE) values of the scale were all above 0.7, 0.8, and 0.6 (Supplementary Table S1), respectively, according to the questionnaire’s results for internal consistency and convergent validity. Factor loadings were ≥ 0.7, CR values > 0.7, and AVE values > 0.5, and each variable’s square root of AVE was greater than the correlation coefficient between the variables (Supplementary Table S2), indicating good validity (46).

### 2.4 Quality control

The investigators involved in this study were uniformly trained before the survey. They introduced the basic information about the purpose and significance of this study to the respondents. The principles of opting out of the survey and protecting the privacy of personal information were also explained to the participants by the study’s researchers. After obtaining the consent of the respondents, the anonymous questionnaires were filled out to collect the information. Respondents filled out the questionnaire voluntarily and independently. The completed questionnaires were checked and recovered immediately on the spot and screened for completeness by the researchers. In this study, a total of 512 questionnaires were completed, all of which were fully completed. Among them, 31 questionnaires were excluded: 11 for excessively short response times, 5 for logical inconsistencies in answers, and 15 for answers with repetitions. Additionally, we examined response consistency in the scale sections, excluding 4 questionnaires with all answers marked as “1,” 8 with all “3,” and 3 with all “5.” The study incorporated a total of 481 valid questionnaires.

### 2.5 Statistical analysis

Excel was used to establish the database, and the data were double-recorded by two people. Data were analyzed descriptively, exploratory factor analysis (EFA), reliability test, and a one-way repeated-measures ANOVA using SPSS software. The social and Demographic information was expressed as frequency and percentage, and measurement information was expressed in the form of (mean ± standard deviation) for the dimensions and mean scores of the entries. AMOS 28.0 software was used to conduct a confirmatory factor analysis (CFA) and common method bias. To construct a structural equation model to validate the establishment of theoretical assumptions. Statistical significance was taken as *p* < 0.05.

### 2.6 Ethical approval

This ethics approval was proved by the Ethics Committee of Chongqing Medical University (reference number: 2025-041).

## 3 Results

### 3.1 Accessibility, social norms, perceived usefulness and vaccination intention

Descriptive statistical analyses were performed on the mean scores for each dimension. The overall accessibility dimension yielded a mean score of 3.38 ± 0.81, social norms averaged 3.72 ± 0.79, and perceived usefulness had a mean score of 3.45 ± 0.75. A one-way repeated-measures ANOVA was conducted to compare the four items within the accessibility dimension. Mauchly’s test of sphericity indicated a violation of the sphericity assumption (*W* = 0.655, *p* < 0.001); consequently, degrees of freedom were adjusted using the Greenhouse–Geisser correction (47). The analysis revealed a significant main effect of item (*F* = 27.69, *p* < 0.001). Subsequent Bonferroni-corrected pairwise comparisons demonstrated that convenience (3.53 ± 0.89) was rated significantly higher than availability (3.42 ± 0.94, *p* = 0.002), acceptance (3.33 ± 0.85, *p* < 0.001), and affordability (3.25 ± 0.98, *p* < 0.001). Similarly, applying the Greenhouse–Geisser correction to the repeated-measures ANOVA for social norms items revealed a significant main effect (*F* = 19.238, *p* < 0.001). Within this dimension, recommendation from medical personnel (3.83 ± 0.91) was rated significantly higher than those from the general public (3.73 ± 0.92, *p* = 0.008) and from family and friends (3.61 ± 0.91, *p* < 0.001). Regarding perceived, confidence of perceived usefulness was rated higher than effectiveness (3.46 ± 0.87, *p* < 0.001), benefits (3.40 ± 0.91, *p* < 0.001), and perceived vaccine safety (3.31 ± 0.87, *p* < 0.001). The total score of intention was 3.39 ± 0.81. Wilcoxon signed-rank test (*Z* = −2.27, *p* = 0.023) indicated that participants’ intention to recommend vaccination (3.42 ± 1.05) was significantly higher than their intention to vaccinate themselves (3.36 ± 1.04).

### 3.2 Structural equation modeling analysis

This study established a path diagram exerting Structural equation modeling to explore the correlation of vaccination service accessibility, social norms, perceived usefulness and vaccination intention. The *χ*^2^/df of the mode is 2.677 (*χ*^2^/df < 3), root mean square error of approximation (RMSEA) is 0.059 < 0.08, goodness-of-fit index (GFI) is 0.954, comparative fit index (CFI) is 0.978, and Tucker-Lewis index (TLI) is 0.970, which were greater than 0.9. To assess common method bias, we employed the unmeasured latent method factor approach. This approach required constructing a confirmatory factor analysis (CFA) model (48). We added a method factor to the original CFA model, allowing all measurement items to load on both their theoretical constructs and the method factor. Results showed that fit index changes were <0.02 compared to the original model, indicating no significant change after adding the common method factor (49).

**Figure 1:**
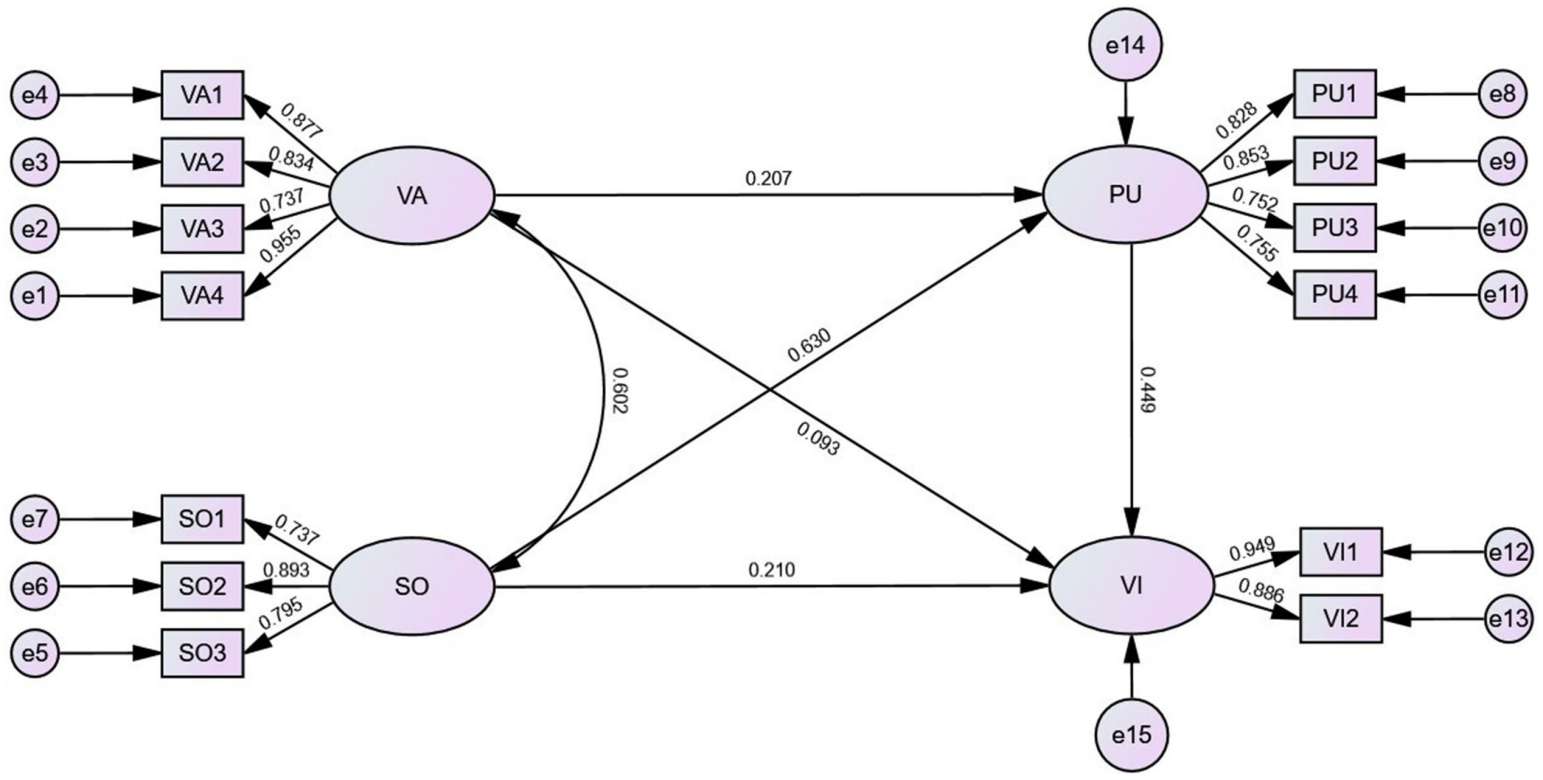
Structural equation modeling of accessibility of vaccination service, social norms, perceived usefulness and vaccination intention (sample size = 481). For path arrows, the digits were standardized regression coefficients. The circles indicated latent variables and boxes represented observed indexes. VA, accessibility of vaccination services; SO, social norms; PU, perceived usefulness; VI, Vaccination intention.

The hypotheses were tested using the Bootstrap method. The results showed that social norms (β = 0.210, *p* = 0.004) and perceived usefulness (β = 0.449, *p <* 0.001) were positively associated with the intention of middle-aged and older adults to be vaccinated against herpes zoster, while the accessibility of vaccination services had no significant association on the willingness to be vaccinated, and the hypotheses H2 and H3 were proven. The accessibility of vaccination services (β = 0.207, *p <* 0.001) and social norms (β = 0.630, *p <* 0.001) showed positive associations with perceived usefulness (Table 3; Figure 1), respectively, and the hypotheses H4 and H5 were proven.

**Table 3:** Structural equation modeling results.

Hypotheses	Paths	Standard regression coefficient	Standard error	*p-*value
H1	Accessibility of vaccination services → vaccination intention	0.093	0.063	0.072
H2	Social norms →vaccination intention	0.210	0.100	0.004
H3	Perceived usefulness →vaccination intention	0.449	0.105	<0.001
H4	Accessibility of vaccination services → perceived usefulness	0.207	0.042	<0.001
H5	Social norms → perceived usefulness	0.630	0.056	<0.001

*H1: Accessibility of vaccination services is positively related to HZV intentions among middle-aged and older adults; H2: Social norms are positively associated with HZV intentions in middle-aged and older adults; H3: Perceived usefulness is positively related to HZV intentions in middle-aged and older adults; H4: Accessibility of vaccination services is positively related to the perceived usefulness of HZV in middle-aged and older adults; H5: Social norms are positively related to perceived usefulness of HZV in middle-aged and older adults; H6: Perceived usefulness of HZV plays a mediating role in the accessibility of vaccination services and vaccination intention; H7: Perceived usefulness of the HZV plays a mediating role in social norms and vaccination intention.

The results of the mediating role test show that the accessibility of vaccination services significantly and positively associated with the intention of middle-aged and older adults to be vaccinated through perceived usefulness (β *=* 0.093, *p <* 0.001), and the assumption that H6 is valid. Social norms showed a significant positive association with the intention through perceived usefulness (β = 0.283, *p <* 0.001). Partial mediation effects exist and hypothesis H7 is proven. The specific results are shown in Table 4.

**Table 4:** Results of the mediation effect test.

Hypotheses	Paths	Standard regression coefficient (95% CI)	Total effect (95% CI)	Direct effect (95% CI)	standard error
H6	Accessibility of vaccination services → perceived usefulness → vaccination intention	0.093*** (0.047–0.161)	0.186 (0.093–0.290)	0.093 (0.004–0.183)	0.033
H7	Social norms → perceived usefulness → vaccination intention	0.283*** (0.198–0.386)	0.493 (0.390–0.590)	0.210 (0.081–0.333)	0.057

*H6: Perceived usefulness of HZV plays a mediating role in the accessibility of vaccination services and vaccination intention; H7: Perceived usefulness of the HZV plays a mediating role in social norms and vaccination intention. ***P < 0.001.

## 4 Discussion

The total score of HZV intention of middle-aged and older adults in this study is 3.39 ± 0.81, which is at a medium level. The result is similar to the results of the study conducted by related research in Beijing (50). But the intention is lower than that found in a study conducted on people aged 50 and above in WHO member states worldwide (7). The intention of adults aged 45 and above in Chongqing to receive the herpes zoster vaccine still needs to be improved. Respondents more likely to recommend HZV vaccination to family members and friends. This phenomenon may be attributed to the mutual transmission of knowledge gained through information exchange in the recommendation process. Messages from socially proximate groups with frequent activity interactions among middle-aged and older adults may influence health behavior adoption (39).

This study suggests that social norms are associated with vaccination intention. Middle-aged and older adults may be more motivated to get vaccine, particularly when vaccination is perceived as a widely endorsed and positive social norm. In our study, social norms scores were highest among medical workers, suggesting that recommendations from healthcare providers help enhance vaccination intention (38, 51, 52). Studies conducted in the United Kingdom revealed that more than 80% of individuals inclined to receive the HZV make their medical decisions in collaboration with their general practitioners. The majority of participants reported acquiring vaccine-related information during routine primary care appointments (30). Conversely, a study conducted in Beijing found that only 9.90% of participants learned about the HZV through a physician’s recommendation (42). Limited access to healthcare professionals has been linked to lower vaccination intentions (53). These findings suggest that community outreach should be expanded to increase HZV awareness and immunization rates among vulnerable groups.

In cross-cultural settings, the guidance provided by healthcare professionals is widely acknowledged as playing a significant role in vaccination uptake (54). This cross-cultural consistency likely stems from a shared mechanisms: trust relationship. For the instance, a recent survey of young Americans has revealed a need for greater trust in medical professionals. The establishment of robust and trusting relationships between medical professionals and adolescents has the potential to facilitate informed decision-making regarding vaccinations among this demographic (55). Even in contexts with high vaccine hesitancy, peoples’ trust in healthcare providers’ expertise consistently mitigated doubts about vaccine safety (56). Healthcare providers are universally perceived as credible sources of health information, given their professional training and role as “trusted intermediaries” between scientific knowledge and the public. And patients’ trust in medical workers is built not only on doctors’ medical knowledge but also through health-care experiences (57). Prior positive health-care experiences generalize to vaccine acceptance. A phenomenon consistently observed in the Chinese context where individuals exhibited stronger trust in familiar healthcare providers. Their interpersonal interactions—whether in clinical consultations or community outreach—allow for tailored communication that addresses concerns (58).

These findings carry critical implications for universal vaccine promotion strategies. For one, they emphasize that enhancing medical workers knowledge of HZV and its vaccination is essential. Continuing medical education and reliable information access constitute critical components for maintaining healthcare providers’ professional confidence and their ability to deliver appropriate vaccine recommendations (59). Additionally, it is imperative for healthcare professionals to emphasize communication skills and to convey straightforward, accurate, and scientifically reliable vaccine-related information to middle-aged and older adults. However, it is important to note that the between social norms and vaccination intention may vary considerably across individualistic cultures and religious communities. In the context of China’s acquaintance-based society, individual health behaviors are substantially influenced by collective consensus and shared beliefs within social networks. Although research in the United States has found a significant increase in vaccination rates following a spouse’s diagnosis of HZ, individuals are generally more susceptible to social norms and family pressures in a collectivist society than those in an individualistic society (60). In Muslim families in Malaysia, parents rejected the human papillomavirus vaccination is often rooted in perceived conflicts with religious beliefs (61). In contrast, the survey in Indonesia showed that 11.3% of parents viewed religion as a facilitator factor in favor of human papillomavirus vaccination (62). Furthermore, the magnitude of effect can be amplified by tailoring the recommendation to local institutional and cultural realities. In collectivist societies, providers might leverage community trust in authority figures by framing vaccination as a “family or community health responsibility,” whereas in individualistic contexts, emphasizing personalized health benefits may resonate more.

Prior to this study, we hypothesized that positive association between service accessibility and vaccination intention, but our findings did not support the hypothesis. Instead, the association between accessibility and intention was mediated by perceived usefulness. This is different from the results of related studies (14). This disparity may be explained in that regional policy variations (the types, quantities and prices of vaccine) and limited understanding of vaccination services among middle-aged and older adults may play a significant role in shaping vaccination disparities. Psychological barriers related to the anticipated pain of vaccination can also offset accessibility (44). The HZV was introduced into the national immunization schedule in the United Kingdom, but vaccination rates have declined in subsequent years (63). The results of the Poland also showed that despite the introduction of reimbursement policies and increased availability of the HZV vaccine in 2024, only 47% of respondents reported being aware of its existence (64). In addition, these findings suggest that it is not enough to promote HZV vaccination to the public through accessibility of vaccination services; other valuable factors must also be considered. The sample size could be expanded and the interaction of multiple variables could be analyzed in future studies. We found that although there was no relationship between accessibility and intention to vaccinate with HZV, perceived usefulness mediated the relationship. This interesting result suggests that increasing HZ vaccination rates by improving accessibility alone may be limited if the issue of usefulness is not addressed.

This study revealed a significant positive association between perceived usefulness of the HZV and vaccination intention. Perceived usefulness was found to mediates the relationship between social norms and HZV vaccination intention. In terms of perceived usefulness, middle-aged and older adults generally believe in the government’s decision-making, but are somewhat skeptical about the safety of vaccines. Middle-aged and older individuals show greater vaccination intention when they are no concerns about the safety and effectiveness of vaccines (65). If people believe that vaccines are useful, they will actively seek out avenues for vaccination. This result explains in part why small improvements in accessibility can increase vaccination intentions, when middle-aged and older adults have high levels of perceived usefulness. The primary and cost-effective way to increase public acceptance of vaccine safety is through public education. However, people are often misled by unofficial misinformation that leads to skepticism and misunderstanding about the value and safety of vaccines (66). Delivering such information through formal avenues can foster a social consensus that vaccination contributes to health protection. Additionally, inconsistent government policies have significantly undermined vaccine confidence (67). The Advisory Committee on Immunization Practices (ACIP) has established evidence-based HZV vaccine communication materials (3) to support patient education and health literacy in the United States. China currently need to establish official policy that recommends or provides guidance on vaccination against HZV.

This findings of the study primarily concern the interaction among critical areas, including policy development, public awareness, and infrastructure enhancement. Improving the vaccination intention of HZV requires a framework involving the government, medical institutions, society and, the general public. Compared with other diseases, HZ is not the primary challenge in China. But the level of policy emphasis affects the generalization of the HZV. It is recommended that nationally standardized vaccination guidelines be developed, alongside the implementation of multichannel dissemination strategies utilizing mainstream media, healthcare provider networks, and community outreach initiatives (16). And policy measures should actively cultivate healthcare workers—including community general practitioners and pharmacists (68)—as credible advocates who can articulate the vaccine’s efficacy and safety through repeated, targeted communication. During publicity, healthcare professionals can enhance the vaccination intention of middle-aged and older adults by conducting targeted outreach directed at their friends and family members (69). In addition to enhancing perceived usefulness, vaccination services must be optimized. In China, HZ vaccination is predominantly administered at designated public immunization sites. Variations in resource distribution and staffing levels may hinder access, thereby negatively impacting vaccine uptake among middle-aged and older populations (51). Policymakers should optimize vaccine allocation based on regional population coverage and actual immunization demand. Furthermore, it is essential to establish multiple appointment channels, both online and offline (14), and to adapt service procedures and management practices to accommodate the diverse needs and preferences of middle-aged and older adults. Furthermore, strengthened management of preventive vaccination services (70) was required to ensure vaccine safety and efficacy and prevent negative public sentiment arising from vaccine-related issues. Service feedback mechanisms were utilized to optimize procedures, enabling middle-aged and older adults to perceive measurable improvements in vaccination service accessibility.

In the case of vaccine hesitancy, an emotional assessment of the vaccine may trump a calculation of objective risks and benefits (71). People make their judgments about vaccines—not only on what they think about them but also on the way they feel about it. In this interpretation, both cognitive assessments and emotional components need to be considered (31). The concept of “organism” in the SOR framework is the same as the conclusion and is highly applicable. This study extends the SOR framework to HZ vaccination among middle-aged and older adults, empirically validating perceived usefulness as a multidimensional “organism” reflecting cognitive-evaluative and affective components. Prior research has predominantly utilized frameworks such as the Knowledge-Attitude-Practice (KAP) model, the Health Belief Model (HBM) (31), or the Theory of Planned Behavior (TPB) (32) to investigate vaccination behaviors. These theories have mainly focused on the relationships between vaccination behaviors and various factors, including vaccine-related knowledge, attitudes (72), self-efficacy, risk perception, and perceived benefits or barriers (42), and so on. Building upon this foundation, we examined the dual roles of vaccination service accessibility and social norms, elucidating the mechanisms through which these factors interact with perceived usefulness and vaccination intention.

Our questionnaire was developed by adapting items from well-established scales and validated measures used in previous studies. The final instrument demonstrated sound structural properties and satisfactory reliability and validity. The tool could be adapted to assess vaccine-related attitudes, barriers, and facilitators beyond the specific group. While initially designed for HZV, the core constructs are transferable to other preventive vaccines. A questionnaire validated in older adults for HZV vaccination can, with minor adjustments, reveal whether these same constructs could, for example, predict uptake in adolescents for HPV. The questionnaire could be deployed repeatedly over time to track changes in attitudes and behaviors—to further explore multiple influences between variables. The results of the questionnaire can be used to compare and analyze the results with other studies to find differences and similar results that complement the results of the study. When scaling the questionnaire, key concerns include different culture and specific group (43)—validate and refine items for cultural, linguistic, and demographic nuances to avoid misalignment. Regularly update content to reflect evolving vaccine landscapes, ensuring broader utility without compromising data integrity.

The limitation are as follows. Our mixed sampling approach ensured efficient data collection across diverse socioeconomic regions of Chongqing. Certain limitations regarding universality should be noted: the selection bias, influenced by local community networks’ accessibility. While convenience sampling requires cautious interpretation, readers can assess applicability to their settings using our reported economic strata, inclusion criteria, and results. Future studies using probability sampling could consider populations in different settings. Additionally, our statistical tests suggest common method bias is not significant; we acknowledge the inherent limitations of self-reported data. The reliance on self-reported data may introduce recall and social desirability biases, potentially affecting the accuracy of responses regarding vaccine awareness and willingness. The questionnaire design should incorporate more reverse-coded items and behavioral measurement items. Another limitation is the lack of longitudinal data, which precludes an assessment of causal relationships or changes in attitudes over time. In addition, the integration of multi-theoretical models should be considered to enrich the study. Utilizing longitudinal data to further explore multiple relationships between variables.

## 5 Conclusion

Based on the actual HZV already available in China, this study focuses on the HZV intention of the middle-aged and older population aged ≥45 years. The total score of HZV intention of middle-aged and older adults in this study is 3.39 ± 0.81. Social norms and perceived usefulness are positively associated with the intention of middle-aged and older adults to receive HZV. The accessibility of vaccination service and social norms are related to middle-aged and older adults’ intention of HZV through perceived usefulness. This study extends the SOR framework to HZ vaccination among adults aged 45 and above. This study contributes to a systematic measurement of the mechanisms of dynamic relationships of variables in the context of vaccination. According to the national guidelines and the actual situation, it is meaningful to focus on the willingness of people over 45 years old for HZV. While maintaining high standards of immunization service delivery, targeted efforts should focus on enhancing middle-aged and older adults’ understanding of HZV through authoritative information channels. Pay attention to the awareness and acceptance of HZV among healthcare workers, and use healthcare workers to widely publicize the vaccine. The government should focus on guiding society to form a social consensus on vaccination for disease prevention.

## Data Availability

The raw data supporting the conclusions of this article will be made available by the authors, without undue reservation.
