# Supplementary material for: Relationship among service accessibility, social norms, herpes zoster vaccination intention of middle-aged and older adults in Chongqing: mediating role of perceived usefulness
Source: Front Public Health. 2025 Sep 26;13:1649455. doi: 10.3389/fpubh.2025.1649455 (PMC12510921; doi:10.3389/fpubh.2025.1649455)
Supplement: Supplementary file 1 [file Data_Sheet_1.ZIP › Supplementary material 1.docx]

Supplementary Material

**Table 1 Reliability and validity of the scale(*N*=481).**

| **Variables** | **Items** | **Factor loading** | **AVE** | **Cronbach´ s Alpha** |
| --- | --- | --- | --- | --- |
| Accessibility of vaccination services(VA) | VA1 | 0.877 | 0.730 | 0.908 |
|  | VA2 | 0.834 |  |  |
|  | VA3 | 0.737 |  |  |
|  | VA4 | 0.955 |  |  |
| Social Norms (SO) | SO1 | 0.737 | 0.658 | 0.846 |
|  | SO2 | 0.893 |  |  |
|  | SO3 | 0.795 |  |  |
| Perceived usefulness(PU) | PU1 | 0.828 | 0.637 | 0.873 |
|  | PU2 | 0.853 |  |  |
|  | PU3 | 0.752 |  |  |
|  | PU4 | 0.755 |  |  |
| Intention of vaccination(VI) | VI1 | 0.949 | 0.843 | 0.913 |
|  | VI2 | 0.886 |  |  |

**Table 2** Distinctive validity of the scale(*N*=481).

| **Variables** | **VA** | **SO** | **PU** | **VI** |
| --- | --- | --- | --- | --- |
| VA | 0.730 |  |  |  |
| SO | 0.602 | 0.658 |  |  |
| PU | 0.587 | 0.755 | 0.637 |  |
| VI | 0.483 | 0.605 | 0.663 | 0.843 |
| AVE square root | 0.854 | 0.811 | 0.798 | 0.918 |
